# Vitamin D receptor rs731236 polymorphism and susceptibility to adolescent idiopathic scoliosis in Brazilian girls

**DOI:** 10.3389/fmed.2026.1970879

**Published:** 2026-10-28

**Authors:** Renato H. Tavares, João Antonio M. Guimarães, Ana Beatriz W. Tavares, Antonio Eulalio P. Araujo-Junior, Gustavo B. L. de Azevedo, Luis Antonio M. MoLiterno, Amanda S. Cavalcanti, Helton Luiz A. Defino, Jessica V. Cardoso, Jamila A. Perini

**Affiliations:** 1 Spine Surgery Center, National Institute of Traumatology and Orthopedics (INTO), Rio de Janeiro, RJ, Brazil; 2 Research Division, INTO, Rio de Janeiro, RJ, Brazil; 3 Endocrinology Unit, Internal Medicine Department, Faculty of Medical Sciences, Rio de Janeiro State University (UERJ), Rio de Janeiro, RJ, Brazil; 4 Department of Orthopedics and Traumatology Surgery, Ribeirão Preto Medical School, University of São Paulo (USP), Ribeirão Preto, SP, Brazil; 5 Research Laboratory of Pharmaceutical Sciences (LAPESF), Rio de Janeiro State University (UERJ), Rio de Janeiro, RJ, Brazil

**Keywords:** bone metabolism, curve progression, spinal deformity, taqI variant, VDR polymorphism, vitamin D receptor

## Abstract

**Background:**

The etiology and pathogenesis of adolescent idiopathic scoliosis (AIS) is thought to involve genetic and environmental factors, including low bone mineral density. This study aimed to evaluate the association between vitamin D receptor gene (*VDR*) polymorphism (rs731236, TaqI), curve magnitude, and anthropometric characteristics in girls with AIS.

**Methods:**

A retrospective case-control study included 175 Brazilian girls with AIS and 175 healthy controls. Genotyping was performed using a validated real-time PCR assay. Associations were evaluated using binary and linear regression models.

**Results:**

The median Cobb angle was 48.7° (11–110°), the majority (*n* = 100, 57.1%) had an indication for surgical treatment (Cobb ≥45°), and 120 (68.6%) were skeletally mature (Risser IV and V). Minor allele frequencies of VDR rs731236 (A > G) polymorphism were 41%, 45%, 38%, and 35.7% for all AIS, Cobb < 45° and Cobb ≥45°, and healthy volunteers, respectively. Among underweight and normal-weight participants, however, the *VDR* GG genotype was associated with AIS susceptibility compared to healthy controls (OR = 4.4; CI95%=1.01–19.3). A positive correlation was observed between the presence of the *VDR* GG genotype and an increase in the Cobb angle of 1.2 degrees for every 1 kg/m^2^ increase in BMI (*P* = 0.035).

**Conclusion:**

The *VDR* rs731236 GG genotype was associated with AIS susceptibility among underweight and normal-weight Brazilian girls, suggesting that anthropometric status may modify the phenotypic expression of genetic susceptibility. These findings are hypothesis-generating and require confirmation in larger studies, with control for genetic ancestry, before any clinical application.

## 1 Introduction

Adolescent idiopathic scoliosis (AIS) is the most common spinal deformity observed during adolescence. It is characterized by complex structural changes affecting the three anatomical planes of the spine and has an estimated worldwide prevalence ranging from 1% to 4% (1). The condition predominantly affects females (2), who are at a greater risk of curve progression (3). Although the deformity remains stable in most patients, curve progression may lead to important clinical consequences, including functional disability and reduced quality of life (4). The etiology of AIS is multifactorial and involves the interaction of genetic and environmental factors (5, 6). Recent genomic studies have supported a polygenic architecture for AIS, involving multiple biological pathways related to the extracellular matrix, skeletal muscle contraction and regulation of gene expression (7), and have shown that approximately one in seven severe cases classified as idiopathic may carry an underlying pathogenic variant (8).

Low bone mineral density (BMD) is a phenotype frequently observed in patients with AIS, particularly in girls, and is considered an important prognostic factor for curve progression (9). In this context, vitamin D has attracted considerable interest due to its essential role in calcium homeostasis, bone mineralization, and musculoskeletal growth (10). Studies have demonstrated lower serum vitamin D levels in AIS patients compared with healthy controls, and vitamin D deficiency has been suggested as a potential contributor to scoliosis progression (11).

To exert its biological effects, the active form of vitamin D (1,25(OH)₂D) binds to the vitamin D receptor (VDR), regulating the expression of several genes involved in bone metabolism, cortical and trabecular bone formation, cellular differentiation, and musculoskeletal development (12). VDR is a nuclear receptor encoded by the *VDR* gene, located on chromosome 12q13.11. The *VDR* gene is highly polymorphic, and the TaqI variant (rs731236) has been investigated in several musculoskeletal conditions (13–17). Genetic polymorphism is the presence of two or more variant forms of a specific DNA sequence among different individuals. The most common type of polymorphism is variation at a single nucleotide, known as a single-nucleotide polymorphism (SNP). A genotype is a scoring of the variants present at a specific location in the genome (locus). It can be represented by the actual DNA sequence, such as in the *VDR* rs731236 SNP: AA, AG or GG. A genotype is defined by the combination of two inherited alleles, one from each parent. Some genotypes contribute to an individual's observable traits, called phenotypes (18). This synonymous polymorphism is located in exon 9 (A > G), near the 3′ untranslated region of the gene and may influence mRNA stability, *VDR* expression, vitamin D signaling, and individual responses to vitamin D supplementation (19).

A meta-analysis showed an association of the *VDR* BsmI variant with AIS susceptibility, particularly in Asian populations, whereas no association was found for ApaI (20). The TaqI variant is located in the same 3′ region of the gene as BsmI and ApaI and is in linkage disequilibrium with them (21). It has also been associated with vitamin D levels, BMD and osteoporosis (16, 22–24). These findings make *VDR* rs731236 a biologically plausible candidate for AIS, a condition in which low BMD is a frequent phenotype and a prognostic factor for curve progression (9). Given the established role of *VDR* in bone metabolism and musculoskeletal development, together with the potential functional relevance and relatively high frequency of the *VDR* rs731236 G allele (37.4% in the global population according to the NCBI dbSNP database), this polymorphism represents a biologically plausible candidate for investigation in AIS. However, there is still no consensus on whether this polymorphism can be used as a genetic marker for musculoskeletal disease. Furthermore, to the best of our knowledge, the *VDR* rs731236 polymorphism has not been evaluated in AIS, as previous studies focused on the BsmI, ApaI and FokI variants (20, 25, 26), and no study has been performed in an admixed population.

Therefore, the present study aimed to evaluate the association between the *VDR* rs731236 (TaqI, A > G) polymorphism and AIS in a Brazilian female population, evaluating its relationship with curve magnitude and anthropometric status. Because previous genetic studies have been conducted predominantly in Asian and Caucasian populations, our findings may provide novel insights into the role of *VDR* polymorphisms in a genetically heterogeneous population. Additionally, we summarized the available evidence regarding the association of the *VDR* rs731236 polymorphism with AIS and other musculoskeletal disorders.

## 2 Materials and methods

### 2.1 Study participants and clinical assessment

A retrospective case-control study was conducted at a referral orthopedic center, including 175 Brazilian girls with AIS and 175 healthy women volunteers. The study was approved by the hospital's Research Ethics Committee (73527617.4.0000.5273) and conducted in accordance with the Declaration of Helsinki. Written informed consent was obtained from all participants and/or their legal guardians prior to enrollment. Baseline sociodemographic and clinical data were collected during participant enrollment between 2018 and 2020. Body mass index (BMI) was calculated from weight and height measurements and classified according to age-specific World Health Organization criteria, as previously described by our group (6). Cases of AIS diagnosed during adolescence (i.e., ≤18 years of age) were included. However, the age information presented in the Results section refers to the age at the time of recruitment into the present study.

Participants were diagnosed with spinal curvatures greater than 10°, according to Cobb angle measurements. The Cobb angle was measured on standing posteroanterior full-spine radiographs according to the method described by Cobb (27), as the angle formed between the superior endplate of the upper end vertebra and the inferior endplate of the lower end vertebra, defined as the most tilted vertebrae at each end of the curve. In patients with double or triple curves, adjacent curves shared the same end vertebra, and the curve with the largest Cobb angle was considered the major curve and used for analysis.

As exclusion criteria, male individuals, patients with incomplete radiographic data, those presenting underlying diseases that could justify non-idiopathic scoliosis (neurological, neuromuscular, or syndromic diseases), and individuals without biological material available for genetic analysis were excluded.

AIS cases were stratified according to the Cobb angle of the major curve, measured on the standing radiograph at the time of enrollment, into two groups: <45° and ≥45°. This threshold was chosen because curves of 45–50° or more are the generally accepted indication for surgical treatment in skeletally immature patients (4, 28, 29), and curves exceeding 40–50° at skeletal maturity tend to continue progressing during adulthood (30–32). Skeletal maturity was assessed using the Risser sign, based on iliac apophysis ossification (33). Because the clinical significance of a given Cobb angle depends on age and skeletal maturity (34), this classification was used as a measure of curve magnitude at the time of assessment and not of curve progression, and age at the time of radiography was considered as potential confounders. All radiographic measurements were performed using Surgimap Spine software (Nemaris Inc., New York, USA), version 2.2.15.5.

The healthy group consisted of 175 volunteers randomly recruited from the hospital blood donor bank between March and December 2017 during routine donation visits. The participants had no history of self-reported musculoskeletal diseases, including scoliosis. The study was approved by the respective Institutional Research Ethics Committees (protocol number 1.467.071), and all participants provided informed consent.

### 2.2 Genotyping analysis

Genomic DNA was extracted from oral mucosa samples using a commercial kit (Qiagen, Hilden, Germany), following the manufacturer's instructions. Briefly, 20 µL of proteinase and 400 µL of lysis buffer were added to a new microtube containing 200 µL of the sample and the mixture was incubated at 56 °C for 20 min. To allow DNA precipitation, the tubes were centrifuged and 400 µL of ethanol was added. Then, 700 µL of the mixture was transferred to a silica column tube, which, after 5 min of centrifugation, retains the DNA. Finally, two washing steps and one elution step of the purified DNA were performed to provide high quality purified DNA.

Genotyping of the *VDR* rs731236 (TaqI, A > G) polymorphism was performed by real-time PCR using a validated TaqMan allelic discrimination assay (C_2404008_10 VIC-FAM probes) on a 7500 Real-Time PCR System (Applied Biosystems, Foster City, CA, USA). To ensure the quality of the genotyping, each SNP reaction included two no-template controls (RNase-free water) and two standardized positive controls for each genotype. Reactions were considered satisfactory if all the positive controls were amplified as expected and there was no amplification in the no-template controls. Genotypes were determined directly by gene counting. The genotyping call rate of the current study was 100%, and there was no missing genotype data.

### 2.3 Statistical analysis

The sample size was estimated *a priori* for a case-control study considering a 95% confidence level, 80% statistical power, and the genotype frequencies reported in previously published studies. Since no association studies investigating the *VDR* rs731236 (TaqI) polymorphism and AIS were identified, the genotype frequencies of a genetically correlated variant in strong linkage disequilibrium with *VDR* rs731236 that was previously evaluated in patients with AIS were used as the reference (20, 21, 25, 26). Based on these estimates, the sample size calculation performed using Epi Info™ version 7.2.7 (Centers for Disease Control and Prevention, Atlanta, GA, USA) indicated a minimum of 151 participants per group, resulting in a total minimum sample size of 302 participants.

The normality of continuous variables was assessed using the Shapiro–Wilk test. As the data did not follow a normal distribution, continuous variables were expressed as median and minimum–maximum values. Categorical variables were presented as absolute (n) and relative (%) frequencies.

Comparisons between groups defined according to Cobb angle magnitude (<45° and ≥45°) were performed using Pearson's chi-square test. Genotype and allele frequencies of the *VDR* rs731236 polymorphism were obtained by direct counting and compared between groups using the same test. The Hardy-Weinberg equilibrium (HWE) for the *VDR* rs731236 polymorphism was determined using a goodness-of-fit test.

The association between the polymorphism and curve magnitude (Cobb angle <45° and ≥45°) was evaluated using binary logistic regression to adjust for potential confounding factors. Results were expressed as odds ratios (ORs) and 95% confidence intervals (95% CIs). Variables with *P* ≤ 0.20 in the univariate analysis were included in the multivariable model, and those with *P* ≤ 0.05 were retained in the final model. Furthermore, Spearman's linear correlation coefficient and linear regression models were used to test the correlation between continuous variables. Statistical analyses were performed using IBM SPSS Statistics version 20.0 (IBM Corp., Armonk, NY, USA), adopting a significance level of 5%.

### 2.4 Literature review

A structured literature review was performed to identify published articles involving the association of the *VDR* rs731236 (TaqI, A > G) polymorphism with musculoskeletal diseases/conditions. The search was conducted in the PubMed database in January 2026 using the following combined keywords: (TaqI polymorphism OR rs731236 polymorphism) AND musculoskeletal diseases. Case-control studies published between January 2015 and December 2025, with full texts available in English and reporting genotype and/or allele frequency data, as well as association results for the *VDR* rs731236 (TaqI, A > G) polymorphism, were included in the review. Review articles, meta-analyses, studies published in languages other than English, and studies without sufficient genotype distribution data were excluded.

The following data were extracted from all selected full-text articles and summarized descriptively: author; publication year; studied population; number of controls; type of musculoskeletal disease/condition; genotype and allele frequency data in controls; and reported association findings. The methodological quality of all included studies was independently evaluated by two reviewers using the STROBE checklist for observational studies, and studies with scores higher than 50% were considered methodologically satisfactory (35).

## 3 Results

### 3.1 Participant characteristics

The median age of the 175 female patients with AIS was 15 years (10–30 years). Among those aged 19 years or older (*n* = 40), the median BMI was 21.5 kg/m^2^ (16.2–39.9), while for patients under 19 years (*n* = 135), the median Z-score for BMI was 0.0 (range = −3.0 to 3.0). All of the participants in the control group were female with a median age of 36 years (16–59) and a median BMI of 26.8 kg/m^2^ (18.4–48.3).

Table 1 shows the categorical variables for all AIS cases and categorized by Cobb angle. Patients with a Cobb angle <45° were younger (median age = 14 years) compared to those with indication of surgical treatment (Cobb ≥45°) (median age = 17 years, *P* < 0.001). The median Cobb angle was 48.7° (11–110°), with 75 patients (42.9%) presenting Cobb angles <45° and 100 patients (57.1%) presenting Cobb angles ≥45°. Regarding skeletal maturity assessed using the Risser method (Figure 1) most patients were in advanced stages (grades IV and V, 68.6%). Patients with Cobb ≥45° were more likely to be in advanced skeletal maturity stages compared to those with Cobb <45° (60% vs. 25.3%, *P* < 0.001).

**Table 1:** Demographic and clinical data of the adolescent idiopathic scoliosis.

Variables	AIS Cases (*n* = 175)	Cobb <45° (*n* = 75)	Cobb ≥ 45° (*n* = 100)	*P*-value^a^
	n (%)			
Age				
10–12	33 (22.7)	17 (90.7)	16 (16.0)	
13–15	62 (48.0)	36 (5.3)	26 (26.0)	<0.001
≥ 16	80 (29.3)	22 (4.0)	58 (58.0)	
BMI (kg/m^2^)^b^				
≤ 18.50	8 (20.0)	2 (33.3)	6 (17.6)	0.03
18.51–24.9	21 (52.5)	1 (16.7)	20 (58.8)	
25.0–29.9	6 (15.0)	3 (50.0)	3 (8.8)	
≥ 30	5 (12.5)	0 (0.0)	5 (14.7)	
BMI-for-age (z-score)^c^				
Underweight	8 (5.9)	4 (5.8)	4 (6.0)	
Eutrophic	101 (74.8)	54 (78.3)	47 (71.2)	0.62
Overweight	19 (14.1)	9 (13.0)	10 (15.2)	
Obese	7 (5.2)	2 (2.9)	5 (7.6)	
Anthropometric categories^d^				
Underweight	16 (9.1)	6 (8.0)	10 (10.0)	0.25
Normal weight	122 (69.7)	55 (73.3)	67 (67.0)	
Overweight	25 (14.3)	12 (16.0)	13 (13.0)	
Obesity	12 (6.9)	2 (2.7)	10 (10.0)	
Risser scale				
0	18 (10.3)	12 (16.0)	6 (6.0)	<0.001
I	12 (6.9)	9 (12.0)	3 (3.0)	
II	9 (5.1)	4 (5.3)	5 (5.0)	
III	16 (9.1)	8 (10.7)	8 (8.0)	
IV	41 (23.5)	23 (30.7)	18 (18.0)	
V	79 (45.1)	19 (25.3)	60 (60.0)	

BMI, body mass index.

a Pearson's Chi-square test (*χ*^2^) or Fisher's exact test, when appropriate.

b BMI was calculated for individuals >19 years of age (*n* = 40).

c The z-score was calculated for individuals ≤19 years of age (*n* = 135).

d BMI for adults and z-scores for children and adolescents were grouped (*n* = 175).

**Figure 1:**
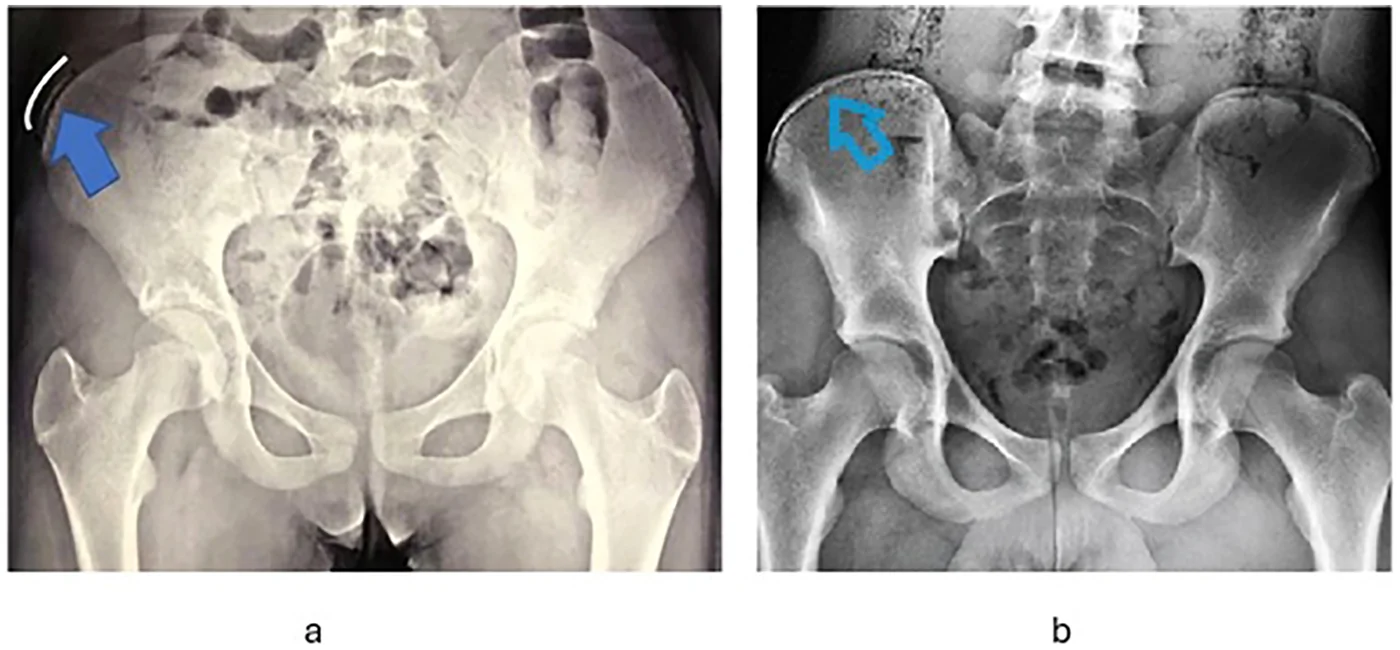
Risser classification. Representative pelvic radiographs illustrate skeletal maturity according to the Risser classification. Scoliosis progression in patients with Risser grades I **(a)** and IV **(b)** are measured using pelvic radiographs, which are based on iliac apophysis ossification.

### 3.2 Distribution and association of the *VDR* rs731236 polymorphism

The genotype distributions of the *VDR* rs731236 (TaqI, A > G) polymorphism were in HWE in both AIS cases and healthy controls (*P* = 0.61 and *P* = 0.16, respectively). The frequencies of the genotypes and alleles of the *VDR* rs731236 (TaqI, A > G) polymorphism were described for all AIS cases, and were then stratified according to curve magnitude (Cobb angle), as illustrated in Figure 2. The *VDR* rs731236 GG genotype was associated with a reduced likelihood of Cobb angle ≥45° in the recessive model (*P* = 0.05) (Table 2). However, after adjusting for potential confounding factors (age and BMI) in logistic regression models, a borderline association was found between scoliosis curve magnitude (Cobb <45° versus Cobb ≥45°) and the *VDR* rs731236 GG genotype.

**Figure 2:**
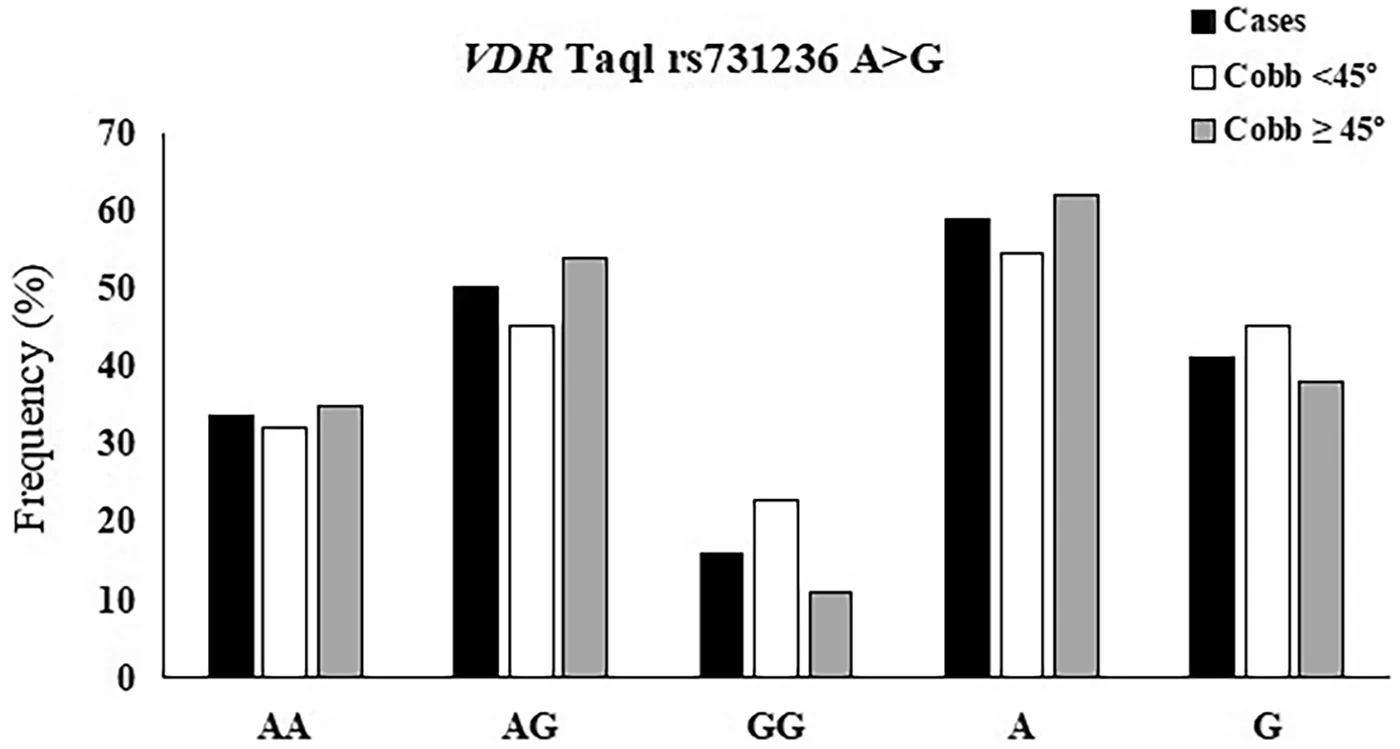
The genotypic and allelic distributions of the *VDR* rs731236 (taqI, A > G) polymorphism in all adolescent idiopathic scoliosis cases, and among curve magnitude groups (cobb angle <45° and ≥45°).

**Table 2:** Association analysis of the *VDR* rs731236 polymorphism in relation to the severity of adolescent idiopathic scoliosis, as measured by the cobb angle.

Genotypes *VDR* TaqI (A > G)	Cobb < 45° (*n* = 75)	Cobb ≥ 45° (*n* = 100)	Crude OR (95% CI)	Adjusted OR^b^ (95% CI)
rs731236				
AA	24 (32.0)	35 (35.0)	1^a^	1^a^
AG	34 (45.3)	54 (54.0)	1.09 (0.55–2.14)	0.84 (0.41–1.71)
GG	17 (22.7)	11 (11.0)	0.44 (0.18–1.11)	0.46 (0.17–1.21)
AA + AG	58 (77.3)	89 (89.0)	1^a^	1^a^
GG	17 (22.7)	11 (11.0)	0.42 (0.18–0.97)	0.41 (0.17–1.01)
A	82 (54.7)	124 (62.0)	1^a^	1^a^
G	68 (45.3)	76 (38.0)	0.74 (0.48–1.14)	0.76 (0.48–1.20)

OR, odds ratio; CI, confidence interval.

a Reference group.

b OR adjusted by age and BMI.

The *VDR* rs731236 GG genotype was associated with AIS among underweight and normal-weight girls (Table 3). After adjusting for the potential confounding factor (age) in logistic regression models, the *VDR* rs731236 GG genotype was found to be associated with an increased susceptibility to AIS in underweight and normal-weight female patients compared with healthy volunteers. No significant association was observed among overweight or obese female participants.

**Table 3:** Association analysis of the *VDR* rs731236 polymorphism between adolescent idiopathic scoliosis patients and healthy volunteers, according to anthropometric categories.

Polymorphism	Underweight/normal weight				
	Healthy volunteers (*n* = 59)	AIS cases (*n* = 138)	*P*-value^a^	OR (95% CI)	Adjusted OR^b^ (95% CI)
*VDR* TaqI rs731236	n (%)				
AA	28 (47.5)	45 (32.6)	**0**.**04**	1^c^	1^c^
AG	28 (47.5)	71 (51.5)		1.58 (0.83–3.00)	0.57 (0.26–1.26)
GG	3 (5.0)	22 (15.9)		**4.56 (1.25–16.7)**	**4.41 (1.01–19.3)**
AA + AG	56 (94.9)	116 (84.1)		1^c^	1^c^
GG	3 (5.1)	22 (15.9)	**0**.**04**	**3.54 (1.02–12.3)**	3.08 (0.65–14.4)

OR, odds ratio, CI, confidence interval.

a *P*-values were calculated using Pearson's chi-square test or Fisher's exact test, when appropriate.

b OR adjusted for age.

c Reference group.

Furthermore, an investigation into the linear relationship between BMI and Cobb angle according to different *VDR* rs731236 genotypes revealed a positive association in the presence of the *VDR* (TaqI, A > G) genotypes (Figure 3). In genotype-stratified analyses, the estimated increase in Cobb angle per 1 kg/m^2^ increase in BMI was 1.2° in the *VDR* GG group (*P* = 0.035) and 0.55° in the *VDR* AA + AG group.

**Figure 3:**
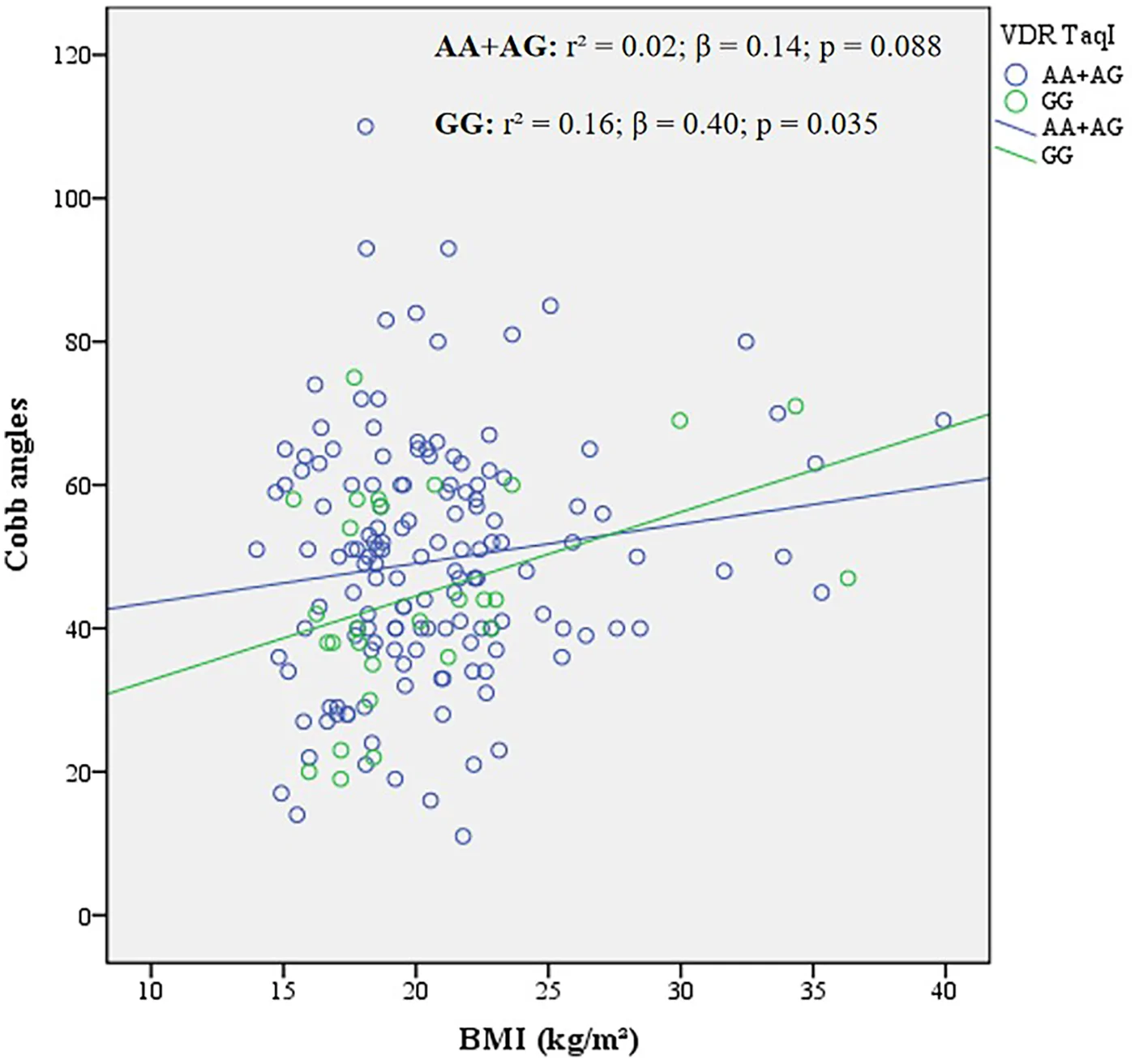
Linear relationship between body mass index (kg/m^2^) and the cobb angle modified by the *VDR* rs731236 (A > G) genotypes. The lines show the linear trend between BMI and the Cobb angle within each genotype group (*VDR* AA + AG versus GG). Adjust R-square for age; Coefficient *β*.

### 3.3 Musculoskeletal disorders risk and *VDR* rs731236 polymorphism: evidence from the literature

A systematic search of the literature on *VDR* rs731236 (TaqI, A > G) polymorphism identified 77 records in the PubMed database. After screening titles and abstracts, 47 (61.0%) studies were excluded based on the following exclusion criteria: 16 (34.0%) review articles or meta-analyses, 8 (17.0%) did not assess the association with musculoskeletal diseases, 4 (8.5%) evaluated genetic polymorphisms in the context of therapeutic response or treatment efficacy rather than disease susceptibility, 2 (4.3%) did not report risk association using OR and 95% CI, 4 (8.5%) studies that did not use a case–control design, and 13 (27.7%) for which full-text access was not available. As a result, 30 studies were selected for full-text review. However, the study by Di Spigna et al. (2016) was excluded because it did not provide sufficient data for risk association analysis. Additionally, 2 relevant studies cited in the reference lists of these articles were included (15, 36), totaling 31 articles in the present literature review.

Table 4 shows the allelic and genotypic frequencies of the *VDR* rs731236 (TaqI, A > G) polymorphism in healthy individuals from various geographical regions around the world. In Asia, the frequency of the *VDR* rs731236 G allele ranged from 1.0% to 61.9% (Chinese, Japanese, Korean, and Taiwanese populations), whereas in European countries it varied from 31.0% to 66.7% (Italians, Poles, Swedes, Britons, Belarusians, Hungarians, Lithuanians, and Ukrainians). In the Middle East and North Africa, contrasting frequencies were observed, ranging from complete absence in Pakistanis to 39.5%–56.0% in Egyptians, with intermediate values in Iranians, Turks, and Tunisians (12.2%–44.6%). In the Americas, Mexicans exhibited a frequency of 24.0 to 35.1%, while Brazilians ranged from 33.0% to 35.7%. The *VDR* rs731236 G allele frequency in the present study of healthy volunteers (35.7%) was similar to reported in most European populations and other Brazilian studies (*P* > 0.05). Among North African and Middle Eastern populations, five of the ten studies analyzed showed comparable frequencies, whereas the remaining studies differed significantly. In contrast, all Asian populations and the Ukrainian population exhibited significantly different allele frequencies compared with the present cohort (*P* < 0.05). Results from Mexican populations were inconsistent, with one study reporting a similar frequency and another showing a significant difference.

**Table 4:** The geographic distribution of the *VDR* rs731236 A > G (taqI) polymorphism worldwide.

Geographic region	Population	Healthy controls (n)	Genotype AG (%)	Genotype GG (%)	MAF (G, %)	^a^*P*-value	References
Asia	Chinese	197	10.7	0.0	5.3	<0.001	Zhang et al., 2017
	Chinese	485	50.7	36.3	61.9	<0.001	Yang et al., 2019
	Chinese	618	7.4	0.2	1.0	<0.001	Cai et al., 2016
	Chinese	200	15.5	2.0	9.8	<0.001	Tao et al., 2018
	Japanese	10	10.0	5.0	10.0	0.02	Yanagawa et al., 2021
	Korean	47	10.6	0.0	5.3	<0.001	Kim et al., 2015
	Taiwanese	600	13.3	1.0	7.7	<0.001	Kuo et al., 2024
Europe	Belarus (postmenopausal)	247	45.8	17.0	39.9	0.22	Marozik et al., 2021
	British (males)	122	55.6	14.5	42.0	0.13	Kow et al., 2019
	Hungarian	93	39.8	19.4	39.0	0.42	Bodoki et al., 2015
	Italian	252	43.2	14.7	36.3	0.86	Colombini et al., 2016
	Italian	73	46.6	19.2	42.5	0.16	Gasperini et al., 2023
	Italian	242	52.5	16.5	42.8	0.04	Latini et al., 2021
	Italian (males)	79	50.6	16.5	41.2	0.24	Cauci et al., 2017
	Lithuanian	180	45.0	9.0	31.0	0.19	Punceviciene et al., 2021
	Polish	39	59.0	7.7	37.2	0.81	Szymczak-Tomczak et al., 2019
	Ukraine	44	40.0	46.7	66.7	<0.001	Ziablitsev et al., 2015
Middle East/North Africa	Arab (postmenopausal)	300	42.0	17.3	39.2	0.29	Ansari et al., 2021
	Egyptian	100	88.0	12.0	56.0	<0.001	Hassan et al., 2022
	Egyptian	50	42.0	36.0	37.0	0.81	Ahmad et al., 2023
	Egyptian	80	48.8	25.0	39.5	0.43	Saad et al., 2015
	Egyptian	45	100.0	0.0	50.0	0.01	Senosi et al., 2022
	Iranian	55	16.4	3.6	12.2	<0.001	Haddadi et al., 2022
	Pakistani	412	0.0	0.0	0.0	<0.001	Mukhtar et al., 2019
	Southeastern Iranian	41	43.9	14.6	36.6	0.88	Yaghoobi et al., 2024
	Tunisian (postmenopausal)	231	39.4	13.3	32.9	0.40	Sassi et al., 2015
	Turkish	146	49.3	19.9	44.6	0.02	Serifoğlu et al., 2024
North America	Mexican Mestizo	114	42.9	2.6	24.0	0.003	González-Huerta et al., 2018
	Mexican Mestizo	204	54.0	8.0	35.1	0.85	Campos-López et al., 2025
South America	Brazilian	112	41.1	12.5	33.0	0.51	Vieira et al., 2018
	Brazilian (athletes)	173	48.4	10.4	34.7	0.78	Marques et al., 2024
	Brazilian^b^	175	50.9	10.3	35.7	1^c^	Present study

MAF, minor allele frequency.

a The *P*-value from the Pearson chi-square test was used to compare the minor allele frequency of the literature with that of the present study.

b Healthy women volunteers were recruited from the blood donor pool at the same hospital where girls with adolescent idiopathic scoliosis were treated.

c Reference population.

Figure 4 shows the scientific publications regarding the association between the *VDR* rs731236 (TaqI, A > G) polymorphism and musculoskeletal disorders. Of the 31 studies included in the review, 17 reported a significant association with an increased risk of musculoskeletal disorders: ankylosing spondylitis (37), low bone mineral density (osteopenia and osteoporosis) with ulcerative colitis (38), degenerative disc disease (14, 15, 39), hallux valgus (17), osteoarthritis (40–42), osteoporosis (16, 22–24), rheumatoid arthritis (13, 41, 43) and osteochondrosis (44, 45). Only two studies, one involving patient with osteopenia (46) and the other involving patients with rheumatoid arthritis (47), reported a reduced risk (OR = 0.68; CI95% 0.51–0.91 and OR = 0.55; CI95% 0.31–0.99, respectively). There were no significant associations between the *VDR* rs731236 polymorphism and the risk of following musculoskeletal conditions: ankylosing spondylitis (48), hallux valgus (49), osteoporosis (50–53), rheumatoid arthritis (36, 54, 55), degenerative disc disease (56), stenosis and/or spondylolisthesis, disc herniation only, discopathies and/or osteochondrosis without herniation (44), traumatic muscle injuries and muscle pain in athletes (57) and idiopathic inflammatory myopathies (58). There are no published studies that have evaluated the presence of the *VDR* rs731236 polymorphism in association with AIS.

**Figure 4:**
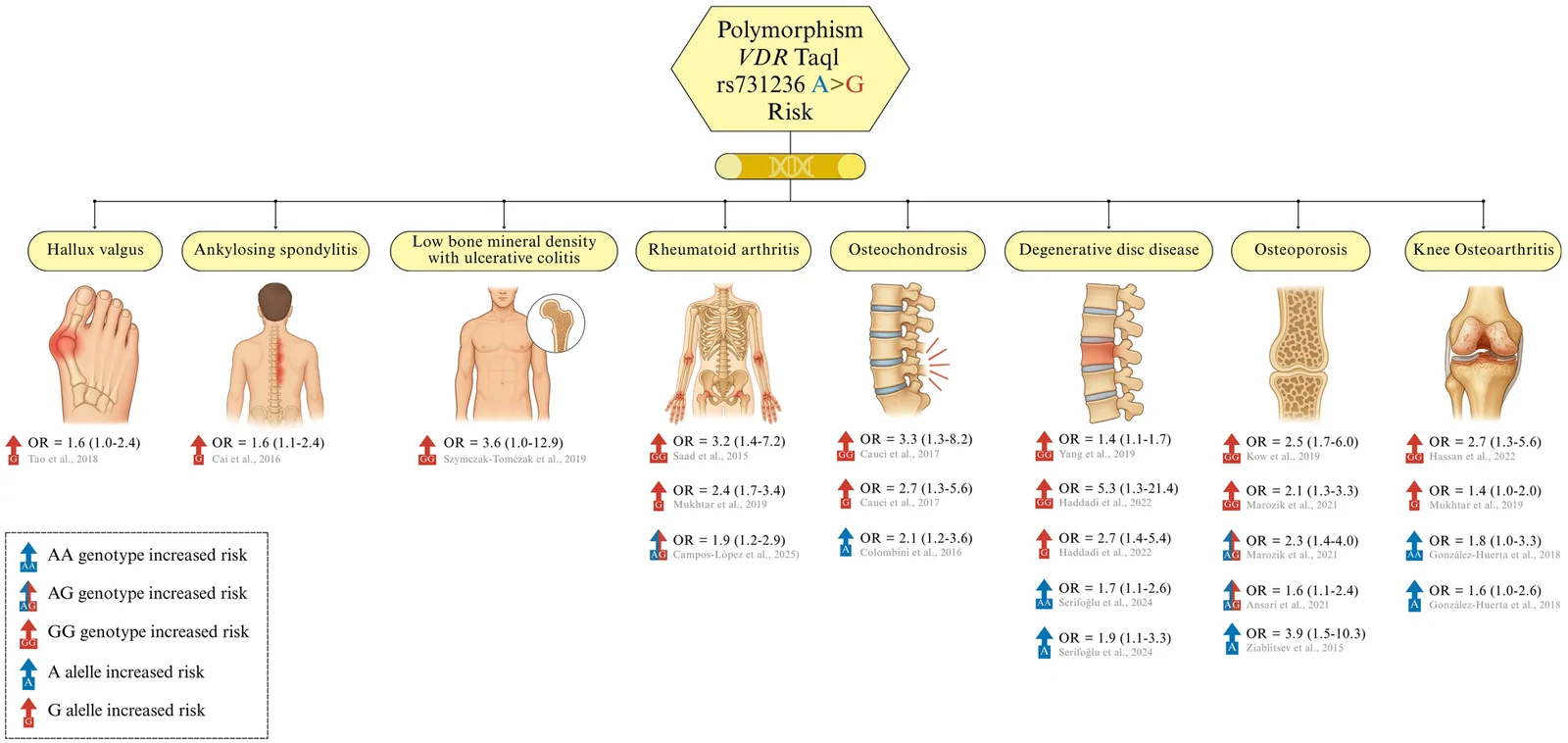
Data from literature regarding risk association between the *VDR* rs731236 (taqI, A > G) polymorphism and musculoskeletal disorders.

## 4 Discussion

The present study investigated the association between *VDR* rs731236 (TaqI, A > G) polymorphism and susceptibility to AIS, curve magnitude, and anthropometric status in 175 Brazilian girls treated at a public orthopedic referral center in a developing country. In addition, the risk association from literature of *VDR* rs731236 polymorphism and musculoskeletal disease was reviewed, since to the best of our knowledge, there are no studies regarding AIS susceptibility and the *VDR* rs731236 polymorphism. Nevertheless, more than half of the available studies reported an association between this polymorphism and an increased risk of musculoskeletal disorders (13–17, 37–45). These include, particularly, osteoporosis, osteoarthritis, degenerative disc disease, rheumatoid arthritis and low bone mineral density. This finding reinforces the potential role of this polymorphism in maintaining skeletal homeostasis, as well as the need to further study this polymorphism in spinal diseases.

From a clinical perspective, the present findings reinforce the concept that AIS should not be regarded solely as a spinal deformity, but rather as a multifactorial condition involving skeletal growth, bone metabolism, body composition, and genetic susceptibility (20). The association observed between the *VDR* rs731236 (TaqI) polymorphism and AIS, particularly among underweight and normal-weight patients, supports the hypothesis that alterations in the vitamin D pathway may contribute to disease expression through mechanisms related to bone mineralization, vertebral growth, and musculoskeletal development. In clinical practice, management of AIS remains largely guided by established parameters such as Cobb angle, skeletal maturity, and growth potential (59). In this context, our findings reveal the association between the *VDR* rs731236 GG genotype and genetic susceptibility to AIS was observed exclusively among underweight and normal-weight girls. This suggests a complex interaction between nutritional status, bone metabolism, and vitamin D signaling. The *VDR* rs731236 (TaqI) polymorphism is located in a region associated with the VDR ligand-binding domain and may affect mRNA splicing, stability, or translational efficiency (60). In addition, it is in linkage disequilibrium with other *VDR* variants, such as BsmI (rs1544410), ApaI (rs7975232), and FokI (rs2228570). These variants are potentially involved in post-transcriptional regulatory mechanisms that affect mRNA stability, expression, and consequently, protein levels (21). Additionally, the *VDR* TaqI (rs731236) polymorphism was associated with vitamin D levels, bone mineral density, and an elevated risk of osteoporosis (61, 62).

It is important to distinguish this susceptibility finding from the curve magnitude analyses: while the case-control comparison (AIS versus healthy controls) revealed a genotype-dependent susceptibility effect modified by BMI, the within-case analyses evaluated curve magnitude (Cobb <45° versus ≥45°) as a marker of disease severity. Therefore, these findings should be interpreted as complementary. Low body weight is a well-recognized phenotype of AIS and has consistently been associated with reduced BMD, delayed menarche, and an increased risk of curve progression (63–65). Moreover, a Mendelian randomization study in a large Japanese cohort showed that genetically determined low BMI has a causal effect on the risk of AIS, with a consistent, although non-significant, trend in a smaller US cohort of European ancestry (66). However, this causal effect of BMI was not observed in a subsequent Mendelian randomization study in European populations (67), suggesting that the relationship between body weight and AIS may differ across populations. Approximately one-third of adolescents with AIS present with osteopenia or low BMD, which has been identified as an independent prognostic factor for curve progression and the need for surgical treatment (9, 64). Genetic evidence also supports a role for bone mass: in European females, genetically estimated higher BMD had a protective causal effect on AIS risk, which was no longer significant after adjustment for BMI, body fat mass and serum 25(OH)D (67), and genetically predicted lower lumbar spine BMD was associated with an increased risk of scoliosis in a Finnish population (68). Taken together, these data are in line with the interplay between nutritional status, vitamin D and genetic susceptibility suggested by our findings. Although studies evaluating circulating vitamin D concentrations in AIS have yielded conflicting results, a recent meta-analysis found no significant differences in serum vitamin D or parathyroid hormone levels between AIS patients and controls, suggesting that alterations in VDR-mediated vitamin D signaling may play a more relevant pathophysiological role (11). Supporting this hypothesis, reduced serum vitamin D levels have been associated with greater curve magnitude in some cohorts (11, 69), while the *VDR* BsmI polymorphism has previously been associated with AIS susceptibility and with lower BMD in AIS patients (20, 25).

Taken together, these findings suggest that the biological effect of the *VDR* rs731236 (TaqI) polymorphism on AIS is modified by a lower BMI. Rather than acting as an isolated risk factor, the association appeared to differ according to anthropometric status, suggesting a potential interaction between the *VDR* genotype and BMI that should be formally evaluated in larger studies. Future prospective studies integrating genetic markers, nutritional status, bone quality measurements, and longitudinal radiographic follow-up may help improve risk stratification for AIS progression. From a public health perspective, given that idiopathic scoliosis affects approximately 1.7% of children and adolescents (2), it is reasonable to ask whether vitamin D supplementation, already recommended for infants to prevent nutritional rickets (70, 71), could play a preventive role. However, evidence in AIS is limited; the lower 25(OH)D levels described in idiopathic scoliosis appear to be environmentally determined (72). Therefore, current evidence does not support supplementation for AIS prevention. Nevertheless, as *VDR* polymorphisms may modify the response to vitamin D supplementation (19), our findings raise the hypothesis that underweight or normal-weight girls carrying the rs731236 GG genotype may deserve particular attention regarding nutritional status and bone health, which should be tested in genotype-stratified prospective studies. To our knowledge, this is the first study to report of the distribution of *VDR* rs731236 (TaqI) polymorphism in AIS cases and in an admixed population. The Brazilian population is the result of extensive admixture from different ancestral roots. Therefore, extrapolating data from other ethnic groups is not appropriate (73). The study examined the reality of treating AIS in a public hospital in a developing country. Additionally, all participants were evaluated by experienced spine surgeons who confirmed the diagnosis and curve magnitude. Multiprofessional approaches, including genetic biomarker analysis, could contribute to more individualized surveillance strategies and potentially improve decision-making regarding observation, brace treatment, and surgical intervention.

### 4.1 Limitations

This study has some limitations: (i) The relatively modest sample size may have limited the precision of the association estimates, as reflected by the wide confidence intervals. In addition, because no previous association studies between *VDR* rs731236 and AIS were available, the sample size calculation was based on estimates derived from a genetically correlated variant in strong linkage disequilibrium (LD) with rs731236 (*VDR* BsmI). Thus, the findings should be interpreted with caution and replicated in larger, independent cohorts; (ii) Curve magnitude was assessed at a single time point, and longitudinal data on curve progression were not available. Although most patients were skeletally mature at the time of assessment and the analyses were adjusted for age, the Cobb angle ≥45° group cannot be interpreted as a marker of progression, and the associations with curve magnitude should be considered exploratory. Moreover, the high proportion of severe AIS cases in our cohort may reflect referral bias related to the specialized nature of the study center. Therefore, the findings may not be directly generalizable to the broader AIS population, particularly patients with milder forms of the condition. (iii) Another limitation regards bone metabolism evaluation. Serum 25-hydroxyvitamin D [25(OH)D], calcium, PTH, bone turnover markers and BMD were not assessed, as they were not part of routine care at our institution. Therefore, the hypothesis that the *VDR* rs731236 polymorphism influences AIS through vitamin D signaling and bone mineralization could not be tested directly. However, a single 25(OH)D measurement reflects recent exposure and is influenced by season, sun exposure, adiposity and outdoor activity, and may not represent vitamin D status during the growth period in which the curve developed. Indeed, the lower 25(OH)D levels reported in idiopathic scoliosis appear to be driven by environmental rather than genetic factors (11, 72), whereas the germline *VDR* genotype is fixed from conception and unaffected by the disease or its treatment. Prospective studies integrating *VDR* genotyping with 25(OH)D, PTH, bone turnover markers and bone densitometry are needed to clarify whether this association is mediated by bone mineral status (74). (iv) Finally, ancestry-informative markers were not available. Therefore, although cases and controls were recruited from the same region and the *VDR* rs731236 allele distribution in controls was similar to that of other healthy Brazilian populations (56, 57), residual confounding by population stratification cannot be excluded in this admixed population (73).

## 5 Conclusion

In summary, the *VDR* rs731236 GG genotype was associated with AIS susceptibility among underweight and normal-weight Brazilian girls. In contrast, associations with curve magnitude were not robust after adjustment and should be considered exploratory. These findings require confirmation in larger, ancestry-controlled studies.

## Data Availability

The datasets presented in this study can be found in online repositories. The names of the repository/repositories and accession number(s) can be found below: https://figshare.com/, DOI: 10.6084/m9.figshare.33269898.
